# Subependymal giant cell astrocytomas in Tuberous Sclerosis Complex have consistent *TSC1/TSC2* biallelic inactivation, and no *BRAF* mutations

**DOI:** 10.18632/oncotarget.20764

**Published:** 2017-09-08

**Authors:** Anika Bongaarts, Krinio Giannikou, Roy J. Reinten, Jasper J. Anink, James D. Mills, Floor E. Jansen, G.M Wim Spliet, Willfred F.A. den Dunnen, Roland Coras, Ingmar Blümcke, Werner Paulus, Theresa Scholl, Martha Feucht, Katarzyna Kotulska, Sergiusz Jozwiak, Anna Maria Buccoliero, Chiara Caporalini, Flavio Giordano, Lorenzo Genitori, Figen Söylemezoğlu, José Pimentel, Mark Nellist, Antoinette Y.N. Schouten-van Meeteren, Anwesha Nag, Angelika Mühlebner, David J. Kwiatkowski, Eleonora Aronica

**Affiliations:** ^1^ Department of (Neuro)Pathology, Academic Medical Center, University of Amsterdam, Amsterdam, The Netherlands; ^2^ Division of Pulmonary and Critical Care Medicine and of Genetics, Brigham and Women's Hospital, Harvard Medical School, Boston, Massachusetts, United States of America; ^3^ Department of Pediatric Neurology, University Medical Center Utrecht, Utrecht, The Netherlands; ^4^ Department of Pathology, University Medical Center Utrecht, Utrecht, The Netherlands; ^5^ Department of Pathology and Medical Biology, University of Groningen, University Medical Center Groningen, Groningen, The Netherlands; ^6^ Department of Neuropathology, University Hospital Erlangen, Erlangen, Germany; ^7^ Institute of Neuropathology, University Hospital Münster, Münster, Germany; ^8^ Department of Pediatrics, Medical University of Vienna, Vienna, Austria; ^9^ Department of Neurology and Epileptology, Children's Memorial Health Institute, Warsaw, Poland; ^10^ Department of Child Neurology, Medical University of Warsaw, Warsaw, Poland; ^11^ Pathology Unit, Anna Meyer Children's Hospital, Florence, Italy; ^12^ Department of Neurosurgery, Anna Meyer Children's Hospital, Florence, Italy; ^13^ Department of Pathology, Faculty of Medicine, Hacettepe University, Ankara, Turkey; ^14^ Department of Neurology, Hospital de Santa Maria, Lisbon, Portugal; ^15^ Department of Clinical Genetics, Erasmus Medical Centre, Rotterdam, The Netherlands; ^16^ Department of Pediatric Oncology, Emma Children's Hospital, Academic Medical Center, University of Amsterdam, Amsterdam, The Netherlands; ^17^ Center for Cancer Genome Discovery, Dana Farber Cancer Institute, Boston, Massachusetts, USA; ^18^ Stichting Epilepsie Instellingen Nederland (SEIN), The Netherlands; ^19^ Swammerdam Institute for Life Sciences, Center for Neuroscience, University of Amsterdam, Amsterdam, The Netherlands

**Keywords:** SEGA, TSC, BRAF, loss of heterozygosity, low grade glioma

## Abstract

Subependymal giant cell astrocytomas (SEGAs) are rare, low-grade glioneuronal brain tumors that occur almost exclusively in patients with tuberous sclerosis complex (TSC). Though histologically benign, SEGAs can lead to serious neurological complications, including hydrocephalus, intractable seizures and death. Previous studies in a limited number of SEGAs have provided evidence for a biallelic two-hit inactivation of either *TSC1 or TSC2*, resulting in constitutive activation of the mechanistic target of rapamycin complex 1 pathway. The activating *BRAF* V600E mutation is a common genetic alteration in low grade gliomas and glioneuronal tumors, and has been reported in SEGAs as well. In the present study, we assessed the prevalence of the *BRAF* V600E mutation in a large cohort of TSC related SEGAs (n=58 patients including 56 with clinical TSC) and found no evidence of either *BRAF* V600E or other mutations in *BRAF.* To confirm that these SEGAs fit the classic model of two hit *TSC1* or *TSC2* inactivation, we also performed massively parallel sequencing of these loci. Nineteen (19) of 34 (56%) samples had mutations in *TSC2*, 10 (29%) had mutations in *TSC1*, while 5 (15%) had no mutation identified in *TSC1*/*TSC2.* The majority of these samples had loss of heterozygosity in the same gene in which the mutation was identified. These results significantly extend previous studies, and in agreement with the Knudson two hit mechanism indicate that biallelic alterations in *TSC2* and less commonly, *TSC1* are consistently seen in SEGAs.

## INTRODUCTION

Subependymal giant cell astrocytomas (SEGAs) are rare, low-grade brain tumors that generally develop during the first two decades of life in 10-20% of patients with tuberous sclerosis complex (TSC) [1–3]. TSC is an autosomal dominant neurocutaneous disorder caused by mutations in either *TSC1* encoding hamartin, or *TSC2* encoding tuberin. Together these two proteins form the TSC protein complex that regulates mechanistic target of rapamycin complex 1 (mTORC1) [4–6]. In the central nervous system, TSC is characterized by the development of SEGAs, subependymal nodules (SEN), cortical tubers and cortical migration tracts [7]. SEGAs represent 1%-2% of all pediatric brain tumors and usually arise near the foramen of Monro [8–10]. They are a potential cause of major morbidity and mortality in TSC [11]. Extended growth of the tumor can cause obstruction of cerebrospinal fluid tract resulting in hydrocephalus and increased intracranial pressure with subsequent death if neglected. SEGAs are treated with either surgical resection or mTORC1 inhibitors including everolimus.

Histopathologically, SEGAs consist of spindle cells, gemistocytic-like cells and giant cells. According to the present world health organization (WHO) classification of brain tumors, SEGAs belong to the group of astrocytic neoplasms, even though they have both glial and neuronal expression patterns [12, 13]. SEGAs likely develop from SEN, but the molecular mechanisms underlying their progressive growth, in contrast to SEN, are unknown so far [14, 15]. There is evidence of second-hit inactivation of *TSC1* or *TSC2* in SEGAs, suggesting that one contributor to SEGA development is the complete loss of a functional tuberin-hamartin complex and the subsequent mTORC1 activation [16–18]. However, it is likely that second-hit mutations in *TSC1* and *TSC2* also contribute to SEN formation, suggesting that additional genetic events may contribute to the progressive growth of SEGAs.

BRAF is a kinase that activates the mitogen-activated protein kinase/extracellular signal-regulated kinase (MAPK/ERK) pathway which regulates cell proliferation, survival and cell-cycle arrest [19]. The *BRAF* c.1799T>A (p.V600E) mutation (*BRAF^V600E^*) results in constitutive activation of MAPK/ERK signaling and is well known in both pediatric and adult low-grade gliomas, including pilocytic astrocytoma (PA), pleomorphic xanthoastrocytoma (PXA), ganglioglioma (GG), desmoplastic infantile gangliogliomas (DIG), and dysembyoplastic neuroepithelial tumor (DNET) [20–26]. Although the prevalence of *BRAF* mutations in low grade gliomas is relatively low [22], *BRAF^V600E^* mutations have been consistently reported as genetic driver in gangliogliomas (18-56%), and have been associated with mTORC1 activation [20, 25].

Both protein kinase B (AKT) and MAPK/ERK pathways have been reported to be activated in SEGAs [27–31]. However, the genetic basis for MAPK/ERK and AKT activation in SEGAs is unknown. The *BRAF^V600E^* mutation was reported in a small set (6 of 14 cases) of SEGAs [23] suggesting that it could explain MAPK/ERK and AKT activation in SEGAs. However, subsequent studies have produced contradictory results, failing to confirm the presence of the *BRAF^V600E^* mutation in SEGAs [18, 21, 23, 26, 32].

In the present study, we examined the possibility that *BRAF* mutations occur in SEGA using a large international cohort of fifty-eight SEGAs from both pediatric and adult TSC patients.

## RESULTS

### Samples and clinical features

Fifty-eight SEGAs and one SEN from 58 patients were analyzed (62% male, 36% female; Table 1). Fifty-six patients had a definite clinical diagnosis of TSC, whereas two patients did not show other signs of TSC apart from the tumor. *TSC1*/*TSC2* mutation analysis was performed as part of routine clinical care on blood or tumor DNA for 19 subjects, such that 7 had *TSC1* and 12 had *TSC2* mutations. For 34 samples we performed *TSC1/TSC2* mutation analysis using massively parallel sequencing (MPS); for the remaining 5 samples there was insufficient DNA for this analysis.

**Table 1:** Summary of clinicopathological features in TSC patients with subependymal giant cell astrocytoma

Parameter	Number	%
Age		
≤18	37	64
>18	21	36
Sex		
Male	36	62
Female	22	37
Tumor location		
Lateral ventricle	49	84
Foramen of Monro	5	9
Third ventricle	4	7
TSC-lesions		
SEN/Tubers	56	96
Tuberous Sclerosis Complex		
Definite	56	97
Possible	2	3

**Figure 1:**
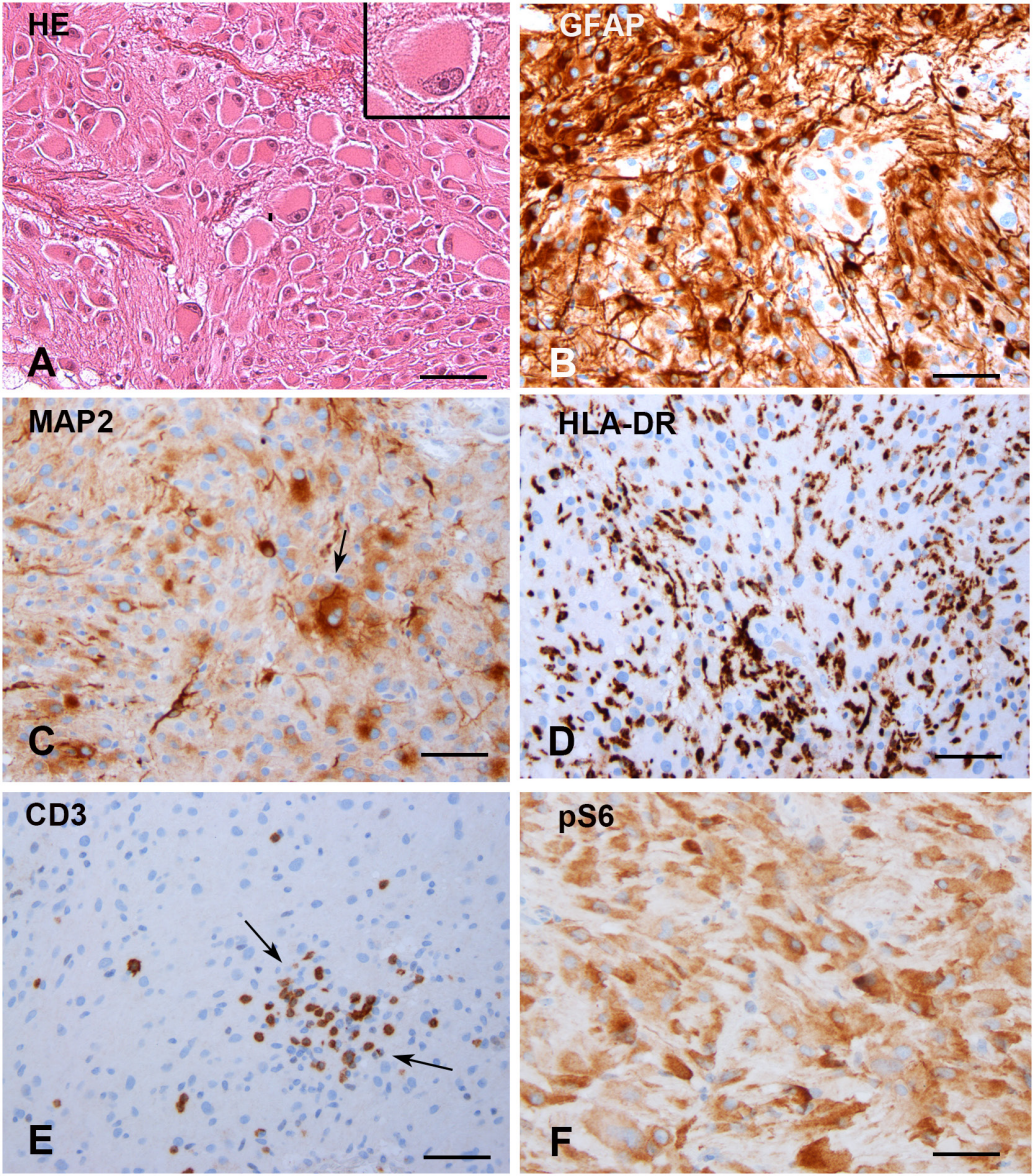
Subependymal giant cell astrocytomas (SEGAs) **(A)** Hematoxylin and eosin staining of a SEGA tumor presenting classical histological features, with giant cells (large cells with abundant eosinophilic cytoplasm and nuclei with prominent nucleoli; high magnification in insert) in a mixed glial background and blood vessels. **(B)** GFAP showing areas of diffuse immunoreactivity. **(C)** Variable expression of neuronal markers, including MAP2 is observed within the tumor (arrow shows MAP2 expression in a giant cell). **(D)** HLA-DR shows prominent presence of microglial cells. **(E)** CD3 staining shows intratumoral T lymphocytes (arrows). **(F)** pS6 shows several positive tumor cells. Scale bars: 80 μm.

Ages ranged from 1 to 53 years at the time of surgery. The large majority of patients had a lesion located in the lateral ventricle near the foramen of Monro and five patients had bilateral tumors. Histological diagnosis was confirmed following the current WHO classification guidelines by two independent neuropathologists [33]. All cases had classical histological features of SEGA, showing mainly giant cells with eosinophilic cytoplasm (Figure 1A). Smaller gemistocytic cells, fibrillary astrocytes and a variable number of multinucleated cells were also noted in all cases. Calcifications were observed in 13/44 FFPE cases (30%). As previously reported [13, 34–36], immunohistochemical analysis revealed variable expression of glial and neuronal markers (Figures 1B-1C). We also observed prominent presence of microglial cells intratumoral T-lymphocytes (Figures 1D-1E) and evidence of activation of mTORC1 pathway with phospho-S6 ribosomal protein immunoreactivity (Figure 1F). The differential diagnosis of SEGA takes into account other tumors arising in the region of the basal ganglia and in the lateral and third ventricles (diffuse astrocytoma, ependymoma, central neurocytoma, choroid plexus papilloma). SEGA outside the setting of TSC are rare [37, 38], as well as SEGA within cortical tubers [39]. In our cohort, nearly all patients (n=56) had other central nervous system TSC-associated lesions (SEN and cortical tubers) associated with refractory epilepsy, making the diagnosis reasonably certain before resection.

### BRAF mutational analysis

**Figure 2:**
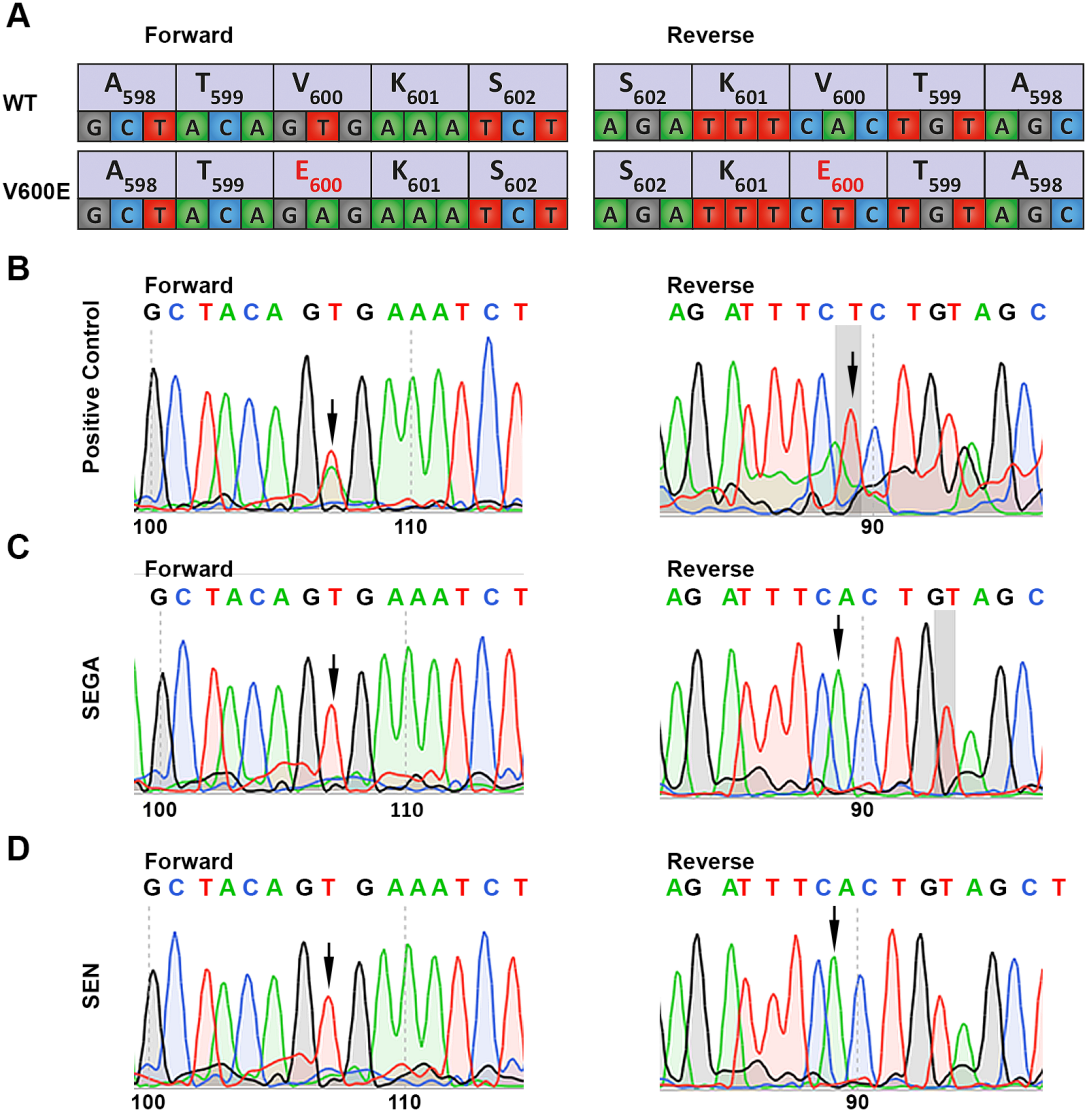
Direct sequencing of exon 15 of *BRAF* for detection of the V600E mutation **(A)** Schematic overview showing the forward and reverse sequence of *BRAF* exon 15 at codon 598 through codon 602 for both wild-type and the c.1799T>A (p.V600E) mutation. **(B)** Positive control. Pilocytic astrocytoma with the *BRAF^V600E^* mutation shows c.1799T>A in the forward sequence (left) and reverse sequence (right), resulting in the p.V600E amino acid substitution (arrow). **(C)** SEGA showing the wild-type GTG forward sequence (left) and CAC reverse sequence (right). **(D)** SEN with the V600 codon showing the wild-type GTG forward sequence (left) and CAC reverse sequence (right). Arrows indicate codon 600 of *BRAF*.

Sanger sequencing analysis for the *BRAF^V600E^* mutation in all 58 SEGA samples tested and the SEN was negative (Figure 2). Furthermore, no other variants were found in exon 15 of *BRAF* in any sample. We also performed RT-PCR to screen for five different types of gene fusions between *KIAA1549* and *BRAF* on 6 SEGAs from which RNA was available (Table 2; data not shown). There was no evidence for the presence of *KIAA1549-BRAF* fusions in the six SEGA cases analyzed. *BRAF* mutational analysis was also performed by MPS for all SEGA samples for which there was sufficient DNA to permit this method of analysis, n=31 (Table 3B). None of the samples showed the *BRAF^V600E^* mutation, even at an allele frequency of 5-10%. Five intronic variants were identified, all known single nucleotide polymorphisms (SNPs; data not shown). Two coding variants in exon 1 were identified, c.82G>T (p.G28C) at allele frequency 100% in one sample, and c.31G>Ap (p.G11S) at allele frequency 56% in a second sample (Table 3B). These are not known germline variants (per Exac). The p.G11S variant has been reported in a single hepatocellular carcinoma, while the p.G28C variant has not been seen previously in cancer (per cBio). Furthermore, these two variants showed no evidence of pathogenicity based on three different in silico prediction tools.

**Table 2:** Primer sequences for detection of *KIAA1549:BRAF* fusion genes

Gene	Exon variant (*KIAA1549:BRAF*)	Forward Primer (5’->3’)	Reverse primer (5’->3’)
*KIAA1549-BRAF* fusion	Ex16:Ex9	CTACAGCCCAGCCCAGAC	GTGAGCCAGGTAATGAGGCAG
*KIAA1549-BRAF* fusion	Ex15:Ex9	CCACAACTCAGCCTACATCGG	GTGAGCCAGGTAATGAGGCAG
*KIAA1549-BRAF* fusion	Ex16:Ex11	AGACGGCCAACAATCCCTGC	GTCCCACTGTAATCTGCCC
*KIAA1549-BRAF* fusion	Ex18:Ex10	GAGGGATCTACTCGGAGGAG	GTGAGCCAGGTAATGAGGCAG
*KIAA1549-BRAF* fusion	Ex19:Ex9	GAAGCGGGGCGAAGAGAG	GTGAGCCAGGTAATGAGGCAG
*PBGD*	-	CTGGTAACGGCAATGCGGCT	GCAGATGGCTCCGATGGTGA
*B2M*	-	AGCATTCAGACTTGTTTCAG	GATGCTGCTTAGATGTCTCG

**Table 3A:** Summary of results for *TSC1/TSC2* mutational analysis in 34 SEGA samples by MPS. NMI = No Mutation Identified, MAF = mutant allele frequency, CN-LOH = Copy neutral loss of heterozygosity, point = point mutation or small insertion or deletion

Case (#)	Gene	Nucleotide change	MAF (%)	Mutation type	Protein change	CN-LOH	Summary
1	NMI						
2	NMI	*TSC1* c.1-7G>A	50	Possible initiator		No	Possible *TSC1* mutation, no CN-LOH
3	*TSC1*	chr9:135700060-135799506del	78	Genomic deletion	deletion of exons 6-23	Yes	large del+ CN-LOH
4	*TSC1*	c.1498C>T	71	Nonsense	p.R500^*^	Yes	point+CN-LOH
5	*TSC1*	c.641_644dupAGAC	93	Insertion	p.F216Dfs^*^3	Yes	point+CN-LOH
6	*TSC1*	c.2074C>T	39	Nonsense	p.R692^*^	Yes	point+CN-LOH
7	*TSC1*	c.1525C>T	12	Nonsense	p.R509^*^	No	Point-no LOH
8	*TSC1*	c.2699dupA	65	Insertion	p.Q901Efs^*^3	Yes	point+CN-LOH
9	*TSC1*	c.1802dupC	79	Insertion	p.P602Sfs^*^4	Yes	point+CN-LOH
10	*TSC1*	c.935dupA	29	Nonsense	p.Y312^*^	Yes	point+CN-LOH
11	*TSC1*	c.1525C>T	76	Nonsense	p.R509^*^	Yes	point+CN-LOH
12	*TSC1*	c.2695C>T	70	Nonsense	p.Q899^*^	Yes	point+CN-LOH
13	*TSC2*	c.4375C>T	50	Nonsense	p.R1459^*^	No	point no LOH (sporadic SEGA; no other signs of TSC)
14	*TSC2*	c.3412C>T	68	Nonsense	p.R1138^*^	Yes	point+CN-LOH
15	*TSC2*	c.2353C>T	12	Nonsense	p.Q785^*^	Yes	point+CN-LOH
16	*TSC2*	c.2221-1G>C	55	Splice	p.L741_splice	Yes	point+CN-LOH
17	*TSC2*	c.790_791delCT	30	Deletion	p.L264Wfs^*^73	No	Point, no LOH
18	*TSC2*	c.903_922delGGCTCTCTGGGGAGCCCACC	34	Deletion	p.W304Ffs^*^27	Yes	point+CN-LOH
19	*TSC2*	c.5227_5244delCGGCTCCGCCACATCAAG	72	In-frame deletion	p.R1743_K1748del	Yes	point+CN-LOH
20	*TSC2*	c.1832G>A	65	Missense	p.R611Q	Yes	point+CN-LOH
21	*TSC2*	c.3526_3527insT	38	Insertion	p.P1176fs	Yes	point+CN-LOH
22	*TSC2*	c.1513C>T	47	Nonsense	p.R505^*^	Yes	point+CN-LOH
23	*TSC2*	c.3171_3172insA	17	Insertion	p.T1059Nfs^*^109	No	point -no LOH
24	*TSC2*	c.268C>T	75	Nonsense	p.Q90^*^	Yes	point+CN-LOH
25	*TSC2*	c.2251C>T	63	Nonsense	p.R751^*^	Yes	point+CN-LOH
26	*TSC2*	c.5227_5244delCGGCTCCGCCACATCAAG	80	In-frame deletion	p.R1743_K1748del	Yes	point+CN-LOH (sporadic SEGA; no other signs of TSC)
27	*TSC2*	c.5168C>A	34	Nonsense	p.S1723^*^	Yes	point+CN-LOH
28	*TSC2*	c.3599G>C	57	Missense	p.R1200P	Yes	point+CN-LOH
29	*TSC2*	c.1372C>T	32	Nonsense	p.R458^*^	Yes	point+CN-LOH
30	*TSC2*	c.3814+1G>C	47	Splice	p.V1272_splice	No	2 points
		c.1831C>T	15	Missense	p.R611W		
31	*TSC2*	c.412G>T	51	Nonsense	p.E138^*^	No	point no LOH
32	*TSC2*					Yes	*TSC2* CN-LOH,no point
33	*TSC2*					Yes	*TSC2* CN-LOH,no point
34	*TSC2*					Yes	*TSC2* CN-LOH,no point

**Table 3B:** Summary of results for *BRAF* mutational analysis by MPS in 31 SEGA samples

Case (#)	Gene	Nucleotide change	MAF (%)	Mutation type	Protein change	Summary
25	*BRAF*	c.82G>T	100	Missense	p.G28C	Novel per cBio,not seen in ExAC
8	*BRAF*	c.31G>A	56	Missense	p.G11S	Seen once in an hepatobiliary cancer (cBio), not seen in ExAC

### TSC1/TSC2 mutational analysis

**Figure 3:**
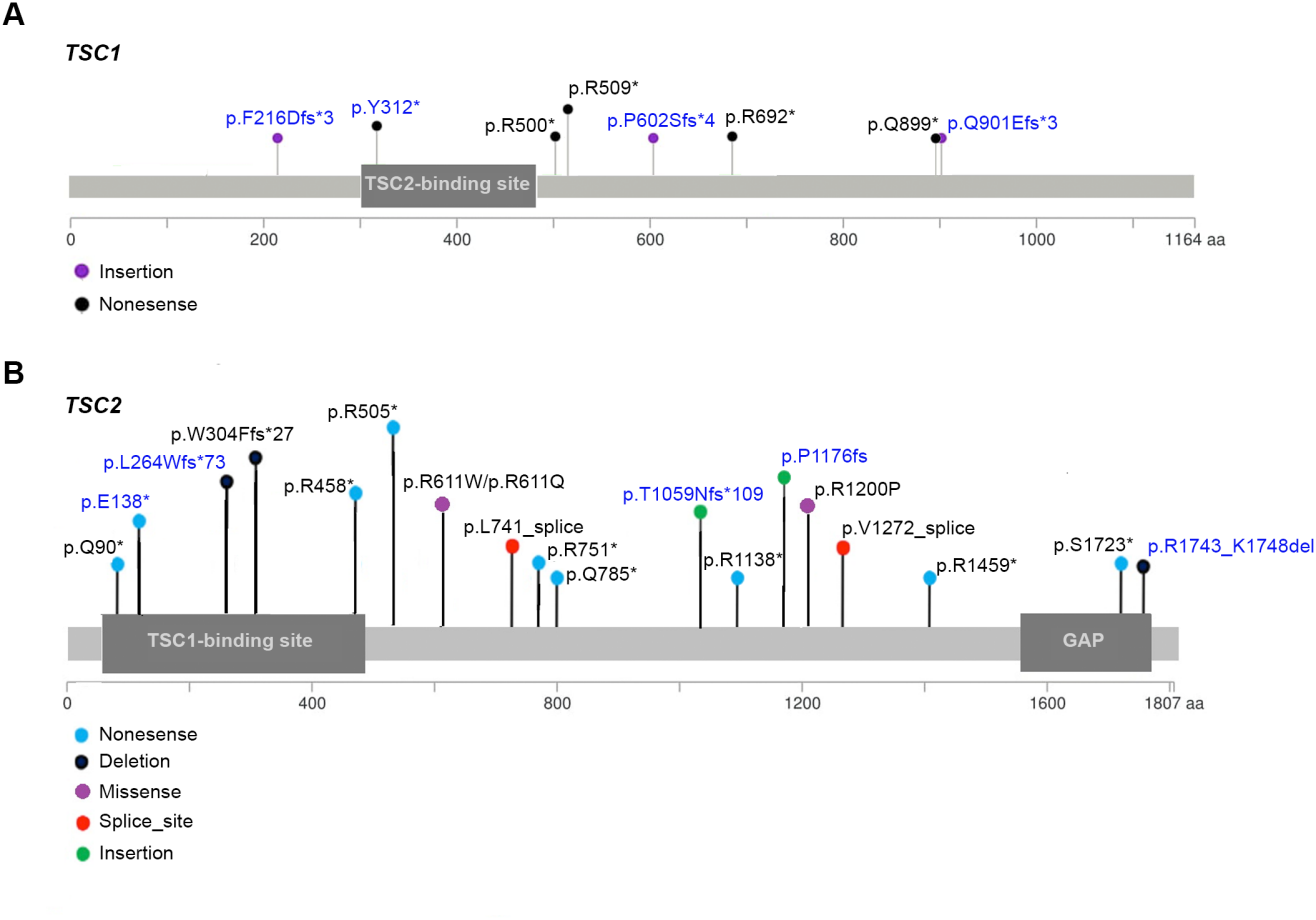
Map of *TSC1* and *TSC2* mutations identified in 10 and 19 SEGA tumors, respectively Novel variants (n = 9) are in blue font whereas variants previously reported (n = 16) are in black font. Circle colors present different mutation types, as indicated. **(A)** Map of TSC1 mutations. One *TSC1* mutation (p.R509^*^) was seen in two different tumor samples; a large genomic deletion (deletion of exons 6-23) and a possible *TSC1* mutation (c.1-7G>A) are not shown. **(B)** map of TSC2 mutations. Two *TSC2* mutations differ by a single nucleotide position in the same amino acid (p.R611Q/ p.R611W), and hence their circles overlap; one *TSC2* mutation (p.R1743_K1748del) was seen in two different tumor samples.

**Figure 4:**
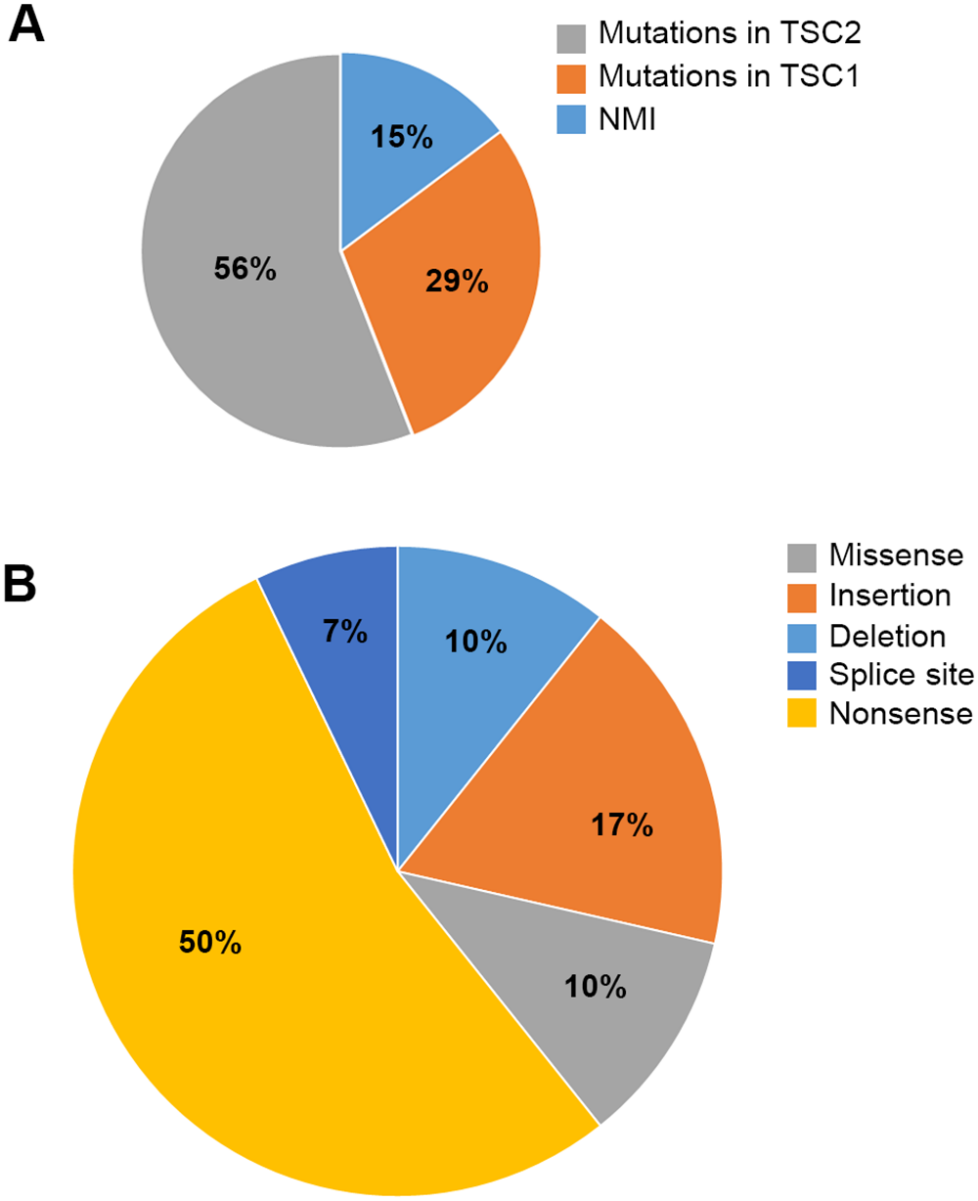
Pie charts demonstrating the *TSC1/TSC2* variant types and mutant allele frequencies in the SEGA tumors analyzed **(A)** Percentage of subjects with *TSC1/TSC2* mutations identified vs. NMI. **(B)** Different mutation types in the SEGA cohort studied.

*TSC1/TSC2* mutational analysis was performed by MPS for 34 SEGA samples (Table 3A, Figures 3 and 4). In 19 (56%) samples *TSC2* mutation was identified, 10 (29%) had mutations in *TSC1*, and 5 (15%) had no mutation identified (NMI) in either *TSC1* or *TSC2.* Of the 5 NMI samples 3 showed copy neutral loss of heterozygosity (CN-LOH) for *TSC2* and another sample had a possible *TSC1* mutation. Nine of 10 (89%) samples with a *TSC1* mutation also showed evidence of CN-LOH for *TSC1*, 14 of 19 (74%) samples with a *TSC2* mutation also showed evidence of CN-LOH for *TSC2*, while in 1 sample two small *TSC2* mutations were identified.

## DISCUSSION

SEGAs are low-grade brain tumors associated with TSC and represent 1%-2% of all pediatric brain tumors [1, 10]. Due to the scarcity of resected SEGAs, studies to investigate the genetic profile of this tumor type have been restricted to a small number of samples/cases. More specifically, investigation of the presence of a *BRAF^V600E^* mutation in SEGAs has been limited to four individual studies with controversial results [18, 23, 26, 32].

In the present study, we analyzed the largest SEGA cohort to date, consisting of fifty-eight SEGAs. Amongst the cohort the vast majority of cases (97%) were clinically diagnosed as definite TSC meeting the required criteria [40, 41]. We did not detect the cancer-actionable *BRAF^V600E^* mutation by direct sequencing or in the MPS analysis in any of the samples tested. Furthermore, there was no evidence for the presence of *KIAA1549-BRAF* fusions in the 6 SEGAs that were analyzed. However, no significant conclusions on *BRAF* fusion mutations in SEGAs could be drawn based on this small sample size (N=6). In the studies that have reported SEGA cases with *BRAF^V600E^* mutations, only two were diagnosed with definite TSC, while the remaining *BRAF^V600E^* positive samples were either TSC negative or defined as possible TSC [23, 26]. Altogether, these results suggest that SEGAs derived from patients with TSC, are negative for the *BRAF^V600E^* mutation [18, 21, 23, 26, 32].

Additionally, our results indicate that *TSC1*/*TSC2* alterations, including CN-LOH, are nearly universally present in SEGAs, consistent with *TSC1*/*TSC2* molecular findings seen in other TSC-related tumors e.g. renal angiomyolipomas (AMLs) and lymphangioleiomyomatosis (LAM) [42]. *TSC2* LOH has also been reported in sporadic renal and hepatic AMLs as well as sporadic perivascular epithelioid cell tumors [42,43]. Conversely, TSC1 mutation and LOH is rare in angiomyolipoma and perivascular epithelioid cell tumors [42–45]. In contrast TSC1 mutations and LOH were relatively common in this series, seen in 10 of 34 (29%) and 9 of 34 (26%), respectively. Regarding the 5 SEGA cases in which no definite small mutation was identified, there are several possible causes. First the DNA quality of many SEGA samples was poor, limiting the sensitivity of the MPS analysis. In particular large genomic deletions may have been missed in this analysis, and are relatively common in TSC2 [42].

Consequently, the mechanism of MAPK/ERK and AKT pathway activation in SEGAs [27–31] is uncertain, and further investigation is required.

## MATERIALS AND METHODS

### SEGA tumor specimens

SEGA specimens were obtained from the following sites: the Academic Medical Center of Amsterdam, the University Medical Center Utrecht, University Medical Center Groningen, University Hospital Erlangen, University Hospital Münster, Medical University of Vienna, Children's Memorial Health Institute in Warsaw, Meyer Children's Hospital in Florence, Hacettepe University in Ankara, and the University Hospital de Santa Maria (CHLN) University Hospital de Santa Maria (CHLN) in Lisbon. Fifty-eight SEGAs and one SEN were available from 58 patients of which 56 met standard diagnostic criteria for TSC (Table 1) [40, 41]. Specimens were obtained and used in accordance with the Declaration of Helsinki and this study was approved by the Medical Ethics Committee of each institution.

### Histopathological evaluation

Tissue was fixed in 10% buffered formalin and embedded in paraffin. Paraffin-embedded tissue was sectioned at 6 μm, mounted on organosilane-coated slides (Sigma, St. Louis, MO, USA) and stained with hematoxylin-eosin (HE) for the morphological evaluation. Histological diagnosis was performed according to the 2016 WHO classification of the central nervous system [33]. Sections of the most representative paraffin-embedded specimen of each case were used for additional immunocytochemical staining, as previously reported [34, 35]. The following antibodies have been used: glial fibrillary acidic protein (GFAP; polyclonal rabbit, DAKO, Glostrup, Denmark; 1:4000; monoclonal mouse; DAKO; 1:50), microtubule-associated protein (MAP2; mouse clone HM2; Sigma 1:100), anti-human leukocyte antigen (HLA)-DP, DQ, DR (mouse clone CR3/43; DAKO; 1:100), CD3 (mouse monoclonal, clone F7.2.38; DAKO; 1:200; T-lymphocytes), phospho-S6 ribosomal protein (Ser235/236; pS6, rabbit polyclonal, Cell Signaling Technology, Beverly, MA, USA; 1:50) and Ki67 (mouse clone MIB-1, DAKO, Glostrup, Denmark. 1:20) were used in the routine immunocytochemical analysis of tumor specimens to document the presence of a heterogeneous population of cells and the activation of the mTORC1 pathway. After washing in PBS, sections were stained with a polymer based peroxidase immunocytochemistry detection kit (BrightVision Peroxidase system, ImmunoVision, Brisbane, CA, USA). Signal was detected using the chromogen 3-amino-9-ethylcarbazole (AEC, Sigma-Aldrich, St. Louis, MO, USA).

### DNA extraction and BRAF^V600E^ mutation analysis

DNA was extracted from both FFPE (n=44) and frozen (n=14) SEGA tumor samples. Since SEGA often display intratumoral hemorrhages, areas of representative tumor (identified on hematoxylin & eosin stained sections) were selected for cases in which hemorrhages, were observed within the FFPE SEGA tissue samples (n=44). Tumor DNA was extracted from 10-μm-thick paraffin sections using BiOstic FFPE Tissue DNA Isolation kit (MO BIO) according to the manufacturer's instructions. From frozen tissue samples (N=14) DNA was recovered from the organic phase following QIAzol (Qiagen) extraction of RNA and was further purified using QIAamp DNA mini Kit (Qiagen). PCR amplification for the entire extent of exon 15 of *BRAF* including codon 600 was performed as previously described using primers TCATAATGCTTGCTCTGATAGGA and GGCCAAAAATTTAATCAGTGGA [26]. Purified PCR products were sequenced by the Sanger method using the Big Dye Terminator Cycle Sequencing Kit (PerkinElmer Biosystems, Foster City, CA, USA).

### KIAA1549–BRAF gene fusion

Six SEGA tissue samples were tested for *KIAA1549*-*BRAF* fusions in a diagnostic setting. Total RNA was extracted from frozen tissue samples using miRNeasy mini kit (Qiagen) according to the manufacturer's instructions. One microgram of total RNA was reverse-transcribed into cDNA, followed by PCR using primer sets corresponding to different *KIAA1549-BRAF* fusion genes and the *PBGD* and *B2M* reference genes (Table 2). PCR products were analyzed on a 2% agarose gel. Pilocytic astrocytoma tissue containing the *KIAA1549*-*BRAF* fusions was used as a positive control. Additionally, tonsil tissue known to lack the *KIAA1549*-*BRAF* fusion genes was used as a negative control.

### TSC1/TSC2 mutation and LOH analysis of SEGAs

In 3 cases (fresh frozen samples), targeted MPS was performed using a HaloPlex custom capture array as described previously [46]. In the other 31 cases (24 FFPE and 7 fresh frozen samples), targeted MPS was performed using a customized gene bait set (Agilent platform) designed in the Kwiatkowski lab that covers the entire *TSC1* and *TSC2* genes including 10 kb upstream and downstream and all coding exons and introns. This bait set also covered all coding exons and adjacent introns of *BRAF*. MPS was performed according to the following methods. Briefly, DNA was subjected to fragmentation using Covaris sonication to an average size of 250bp. The fragmented DNA was purified using Agencourt AMPure XP beads and ligated to the dual indexed adaptors for Illumina sequencing. A MiSeq run was performed to quantify each library. Libraries were then pooled in equal mass and captured using the custom baitset using Agilent SureSelect hybrid capture kit. The captured libraries were then sequenced on the either the HiSeq2500 or the HiSeq 3000 instrument.

The sequencing output was de-convoluted into individual sample reads and sorted using Picard tools [47]. Reads were aligned to the reference sequence hg19 from the Human Genome Reference Consortium using bwa [42, 48–50], and duplicate reads were identified and marked using the Picard tools. The alignments were further refined using the GATK tool for localized realignment around indel sites and recalibration of the quality scores was also performed using GATK tools [42, 49, 51]. Mutation analysis for single nucleotide variants (SNV) was performed using MuTect v1.1.4 and annotated by Variant Effect Predictor (VEP) [52, 53]. Insertions and deletions were called using Indel Locator and SomaticIndelDetector tool [42, 54]. MuTect was run in paired mode using a CEPH sample as a normal since normal DNA samples were not available, and a germline variant filter was then applied. Variants were filtered against the 6,500 exome release of the Exome Sequencing Project (ESP) database ExAC (exclude variants seen in more than 3 normal subjects; http://exac.broadinstitute.org), 1000G and GnomAD [55, 56]. Variants represented at >1% in either the African-American or European-American subsets of these reference databases and not in COSMIC > 2x were considered to be germline. Variants found in *BRAF* were analysed using cBio (http://www.cbioportal.org) and were further assessed for functionality using 3 different in silico prediction tools: PROVEAN (http://provean.jcvi.org), SIFT (http://sift.jcvi.org) and MutationAccessor (http://mutationassessor.org) [57–61].

A second approach was used in parallel to analyze the sequence data, with capture of read calls at all positions using SAMtools Pileup, followed by custom processing in Python and Matlab to determine base call frequency at each position in each read orientation. These data were then filtered to eliminate variant calls observed in only a single read orientation, or seen in multiple samples to exclude artifacts derived from the sequencing process. All variants observed at a frequency of >1% were directly reviewed using the Integrative Genomics Viewer, to identify bona fide variant calls and exclude sequencing or alignment artifacts [21, 23, 26]. Potential pathogenic variants seen at frequency > 1% were also examined in the GnomAD database and the TSC LOVD database.

A minimal median read depth of 20x coverage for the coding exons of *TSC1* and *TSC2* was required for the samples reported here. The median read depth for coding exons of *TSC1* and *TSC2* was a median of 107 (range 20 – 1120) among the 31 samples.

LOH was assessed using two allele frequencies: 1) at the site of mutation, using Unix grep to precisely quantify mutant vs. wild-type reads for indel mutations; and 2) at all SNPs identified in the *TSC1* and *TSC2* genes that had a population allele frequency of > 0.05% in the GnomAD database. If either the mutant allele frequency for the mutation was > 55%, or the median SNP minor allele frequency for *TSC1*/*TSC2* was < 40%, this was considered evidence of CN-LOH. LOH was assessed only in the tumor samples; normal brain tissue adjacent to the tumor, was not available.

## Abbreviations

AKT: protein kinase B
AMLs: angiomyolipomas
*BRAF^V600E^*: *BRAF* V600E mutation
CN-LOH: copy neutral loss of heterozygosity
DIG: desmoplastic infantile gangliogliomas
DNET: dysembyoplastic neuroepithelial tumor
ESP: the Exome Sequencing Project
GFAP: glial fibrillary acidic protein
GG: ganglioglioma
HE: hematoxylin-eosin
HLA: human leukocyte antigen
LAM: lymphangioleiomyomatosis
LOH: loss of heterozygosity
MAF: mutant allele frequency
MAP2: microtubule-associated protein
MAPK/ERK: mitogen-activated protein kinase/extracellular signal-regulated kinase
MPS: massively parallel sequencing
mTORC1: mammalian TOR Complex 1
NMI: no mutation identified
PA: pilocytic astrocytoma
pS6: phospho-S6 ribosomal protein
PXA: pleomorphic xanthoastrocytoma
SEGAs: subependymal giant cell astrocytomas
SEN: subependymal nodules
SNPs: single nucleotide polymorphisms
SNV: single nucleotide variants
TSC: tuberous sclerosis complex
VEP: Variant Effect Predictor
WHO: world health organization

## References

[1] Adriaensen ME, Schaefer-Prokop CM, Stijnen T, Duyndam DA, Zonnenberg BA, Prokop M (2009). Prevalence of subependymal giant cell tumors in patients with tuberous sclerosis and a review of the literature. Eur J Neurol.

[2] Dabora SL, Jozwiak S, Franz DN, Roberts PS, Nieto A, Chung J, Choy YS, Reeve MP, Thiele E, Egelhoff JC, Kasprzyk-Obara J, Domanska-Pakiela D, Kwiatkowski DJ (2001). Mutational analysis in a cohort of 224 tuberous sclerosis patients indicates increased severity of TSC2, compared with TSC1, disease in multiple organs. Am J Hum Genet.

[3] DiMario FJ (2004). Brain abnormalities in tuberous sclerosis complex. J Child Neurol.

[4] Kwiatkowski DJ (2003). Rhebbing up mTOR: new insights on TSC1 and TSC2, and the pathogenesis of tuberous sclerosis. Cancer Biol Ther.

[5] van Slegtenhorst M, de Hoogt R, Hermans C, Nellist M, Janssen B, Verhoef S, Lindhout D, van den Ouweland A, Halley D, Young J, Burley M, Jeremiah S, Woodward K (1997). Identification of the tuberous sclerosis gene TSC1 on chromosome 9q34. Science.

[6] European Chromosome 16 Tuberous Sclerosis C (1993). Identification and characterization of the tuberous sclerosis gene on chromosome 16. Cell.

[7] Mizuguchi M, Takashima S (2001). Neuropathology of tuberous sclerosis. Brain Dev.

[8] Roth J, Roach ES, Bartels U, Jozwiak S, Koenig MK, Weiner HL, Franz DN, Wang HZ (2012). Subependymal giant cell astrocytoma: diagnosis, screening, and treatment. Recommendations from the International Tuberous Sclerosis Complex Consensus Conference.

[9] Cuccia V, Zuccaro G, Sosa F, Monges J, Lubienieky F, Taratuto AL (2003). Subependymal giant cell astrocytoma in children with tuberous sclerosis. Childs Nerv Syst.

[10] Jozwiak S, Mandera M, Mlynarski W (2015). Natural History and Current Treatment Options for Subependymal Giant Cell Astrocytoma in Tuberous Sclerosis Complex. Semin Pediatr Neurol.

[11] de Ribaupierre S, Dorfmuller G, Bulteau C, Fohlen M, Pinard JM, Chiron C, Delalande O (2007). Subependymal giant-cell astrocytomas in pediatric tuberous sclerosis disease: when should we operate?. Neurosurgery.

[12] Bonnin JM, Rubinstein LJ, Papasozomenos SC, Marangos PJ (1984). Subependymal giant cell astrocytoma. Significance and possible cytogenetic implications of an immunohistochemical study. Acta Neuropathol.

[13] Buccoliero AM, Franchi A, Castiglione F, Gheri CF, Mussa F, Giordano F, Genitori L, Taddei GL (2009). Subependymal giant cell astrocytoma (SEGA): Is it an astrocytoma? Morphological, immunohistochemical and ultrastructural study. Neuropathology.

[14] Fujiwara S, Takaki T, Hikita T, Nishio S (1989). Subependymal giant-cell astrocytoma associated with tuberous sclerosis. Do subependymal nodules grow?. Childs Nerv Syst.

[15] Morimoto K, Mogami H (1986). Sequential CT study of subependymal giant-cell astrocytoma associated with tuberous sclerosis. Case report. J Neurosurg.

[16] Chan JA, Zhang H, Roberts PS, Jozwiak S, Wieslawa G, Lewin-Kowalik J, Kotulska K, Kwiatkowski DJ (2004). Pathogenesis of tuberous sclerosis subependymal giant cell astrocytomas: biallelic inactivation of TSC1 or TSC2 leads to mTOR activation. J Neuropathol Exp Neurol.

[17] Henske EP, Wessner LL, Golden J, Scheithauer BW, Vortmeyer AO, Zhuang Z, Klein-Szanto AJ, Kwiatkowski DJ, Yeung RS (1997). Loss of tuberin in both subependymal giant cell astrocytomas and angiomyolipomas supports a two-hit model for the pathogenesis of tuberous sclerosis tumors. Am J Pathol.

[18] Martin KR, Zhou W, Bowman MJ, Shih J, Au KS, Dittenhafer-Reed KE, Sisson KA, Koeman J, Weisenberger DJ, Cottingham SL, DeRoos ST, Devinsky O, Winn ME (2017). The genomic landscape of tuberous sclerosis complex. Nat Commun.

[19] Peyssonnaux C, Eychene A (2001). The Raf/MEK/ERK pathway: new concepts of activation. Biol Cell.

[20] Blumcke I, Aronica E, Becker A, Capper D, Coras R, Honavar M, Jacques TS, Kobow K, Miyata H, Muhlebner A, Pimentel J, Soylemezoglu F, Thom M (2016). Low-grade epilepsy-associated neuroepithelial tumours - the 2016 WHO classification. Nat Rev Neurol.

[21] Brandner S, von Deimling A (2015). Diagnostic, prognostic and predictive relevance of molecular markers in gliomas. Neuropathol Appl Neurobiol.

[22] Brat DJ, Verhaak RG, Aldape KD, Yung WK, Salama SR, Cooper LA, Rheinbay E, Miller CR, Vitucci M, Morozova O, Robertson AG, Noushmehr H, Laird PW (2015). Cancer Genome Atlas Research Network. Comprehensive, Integrative Genomic Analysis of Diffuse Lower-Grade Gliomas. N Engl J Med.

[23] Lee D, Cho YH, Kang SY, Yoon N, Sung CO, Suh YL (2015). BRAF V600E mutations are frequent in dysembryoplastic neuroepithelial tumors and subependymal giant cell astrocytomas. J Surg Oncol.

[24] Penman CL, Faulkner C, Lowis SP, Kurian KM (2015). Current Understanding of BRAF Alterations in Diagnosis, Prognosis, and Therapeutic Targeting in Pediatric Low-Grade Gliomas. Front Oncol.

[25] Prabowo AS, Iyer AM, Veersema TJ, Anink JJ, Schouten-van Meeteren AY, Spliet WG, van Rijen PC, Ferrier CH, Capper D, Thom M, Aronica E (2014). BRAF V600E mutation is associated with mTOR signaling activation in glioneuronal tumors. Brain Pathol.

[26] Schindler G, Capper D, Meyer J, Janzarik W, Omran H, Herold-Mende C, Schmieder K, Wesseling P, Mawrin C, Hasselblatt M, Louis DN, Korshunov A, Pfister S (2011). Analysis of BRAF V600E mutation in 1,320 nervous system tumors reveals high mutation frequencies in pleomorphic xanthoastrocytoma, ganglioglioma and extra-cerebellar pilocytic astrocytoma. Acta Neuropathol.

[27] Han S, Santos TM, Puga A, Roy J, Thiele EA, McCollin M, Stemmer-Rachamimov A, Ramesh V (2004). Phosphorylation of tuberin as a novel mechanism for somatic inactivation of the tuberous sclerosis complex proteins in brain lesions. Cancer Res.

[28] Jozwiak J, Grajkowska W, Kotulska K, Jozwiak S, Zalewski W, Zajaczkowska A, Roszkowski M, Slupianek A, Wlodarski P (2007). Brain tumor formation in tuberous sclerosis depends on Erk activation. Neuromolecular Med.

[29] Ma L, Chen Z, Erdjument-Bromage H, Tempst P, Pandolfi PP (2005). Phosphorylation and functional inactivation of TSC2 by Erk implications for tuberous sclerosis and cancer pathogenesis. Cell.

[30] Ma L, Teruya-Feldstein J, Bonner P, Bernardi R, Franz DN, Witte D, Cordon-Cardo C, Pandolfi PP (2007). Identification of S664 TSC2 phosphorylation as a marker for extracellular signal-regulated kinase mediated mTOR activation in tuberous sclerosis and human cancer. Cancer Res.

[31] Tee AR, Anjum R, Blenis J (2003). Inactivation of the tuberous sclerosis complex-1 and -2 gene products occurs by phosphoinositide 3-kinase/Akt-dependent and -independent phosphorylation of tuberin. J Biol Chem.

[32] Hang JF, Hsu CY, Lin SC, Wu CC, Lee HJ, Ho DM (2017). Thyroid transcription factor-1 distinguishes subependymal giant cell astrocytoma from its mimics and supports its cell origin from the progenitor cells in the medial ganglionic eminence. Mod Pathol.

[33] Louis DN, Perry A, Reifenberger G, von Deimling A, Figarella-Branger D, Cavenee WK, Ohgaki H, Wiestler OD, Kleihues P, Ellison DW (2016). The 2016 World Health Organization Classification of Tumors of the Central Nervous System: a summary. Acta Neuropathol.

[34] Boer K, Troost D, Timmermans W, Gorter JA, Spliet WG, Nellist M, Jansen F, Aronica E (2008). Cellular localization of metabotropic glutamate receptors in cortical tubers and subependymal giant cell tumors of tuberous sclerosis complex. Neuroscience.

[35] Boer K, Jansen F, Nellist M, Redeker S, van den Ouweland AM, Spliet WG, van Nieuwenhuizen O, Troost D, Crino PB, Aronica E (2008). Inflammatory processes in cortical tubers and subependymal giant cell tumors of tuberous sclerosis complex. Epilepsy Res.

[36] Buccoliero AM, Caporalini C, Giordano F, Mussa F, Scagnet M, Moscardi S, Baroni G, Genitori L, Taddei GL (2016). Subependymal giant cell astrocytoma: a lesion with activated mTOR pathway and constant expression of glutamine synthetase. Clin Neuropathol.

[37] Yamamoto K, Yamada K, Nakahara T, Ishihara A, Takaki S, Kochi M, Ushio Y (2002). Rapid regrowth of solitary subependymal giant cell astrocytoma--case report. Neurol Med Chir (Tokyo).

[38] Takei H, Adesina AM, Powell SZ (2009). Solitary subependymal giant cell astrocytoma incidentally found at autopsy in an elderly woman without tuberous sclerosis complex. Neuropathology.

[39] Katz JS, Frankel H, Ma T, Zagzag D, Liechty B, Zeev BB, Tzadok M, Devinsky O, Weiner HL, Roth J (2017). Unique findings of subependymal giant cell astrocytoma within cortical tubers in patients with tuberous sclerosis complex: a histopathological evaluation. Childs Nerv Syst.

[40] Northrup H, Krueger DA (2013). International Tuberous Sclerosis Complex Consensus G. Tuberous sclerosis complex diagnostic criteria update: recommendations of the 2012 Iinternational Tuberous Sclerosis Complex Consensus Conference. Pediatr Neurol.

[41] Gomez M, Sampson J, eds Whittemore V (1999). The Tuberous Sclerosis Complex.

[42] Giannikou K, Malinowska IA, Pugh TJ, Yan R, Tseng YY, Oh C, Kim J, Tyburczy ME, Chekaluk Y, Liu Y, Alesi N, Finlay GA, Wu CL (2016). Whole Exome Sequencing Identifies TSC1/TSC2 Biallelic Loss as the Primary and Sufficient Driver Event for Renal Angiomyolipoma Development. PLoS Genet.

[43] Pan CC, Chung MY, Ng KF, Liu CY, Wang JS, Chai CY, Huang SH, Chen PC, Ho DM (2008). Constant allelic alteration on chromosome 16p (TSC2 gene) in perivascular epithelioid cell tumour (PEComa): genetic evidence for the relationship of PEComa with angiomyolipoma. J Pathol.

[44] Henske EP, Scheithauer BW, Short MP, Wollmann R, Nahmias J, Hornigold N, van Slegtenhorst M, Welsh CT, Kwiatkowski DJ (1996). Allelic loss is frequent in tuberous sclerosis kidney lesions but rare in brain lesions. Am J Hum Genet.

[45] Qin W, Bajaj V, Malinowska I, Lu X, MacConaill L, Wu CL, Kwiatkowski DJ (2011). Angiomyolipoma have common mutations in TSC2 but no other common genetic events. PLoS One.

[46] Nellist M, Brouwer RW, Kockx CE, van Veghel-Plandsoen M, Withagen-Hermans C, Prins-Bakker L, Hoogeveen-Westerveld M, Mrsic A, van den Berg MM, Koopmans AE, de Wit MC, Jansen FE, Maat-Kievit AJ (2015). Targeted Next Generation Sequencing reveals previously unidentified TSC1 and TSC2 mutations. BMC Med Genet.

[47] http://broadinstitute.github.io/picard/picard-metric-definitions.html.

[48] Li H, Durbin R (2009). Fast and accurate short read alignment with Burrows-Wheeler transform. Bioinformatics.

[49] Tyburczy ME, Dies KA, Glass J, Camposano S, Chekaluk Y, Thorner AR, Lin L, Krueger D, Franz DN, Thiele EA, Sahin M, Kwiatkowski DJ (2015). Mosaic and Intronic Mutations in TSC1/TSC2 Explain the Majority of TSC Patients with No Mutation Identified by Conventional Testing. PLoS Genet.

[50] http://bio-bwa.sourceforge.net/bwa.shtml.

[51] McKenna A, Hanna M, Banks E, Sivachenko A, Cibulskis K, Kernytsky A, Garimella K, Altshuler D, Gabriel S, Daly M, DePristo MA (2010). The Genome Analysis Toolkit: a MapReduce framework for analyzing next-generation DNA sequencing data. Genome Res.

[52] Cibulskis K, Lawrence MS, Carter SL, Sivachenko A, Jaffe D, Sougnez C, Gabriel S, Meyerson M, Lander ES, Getz G (2013). Sensitive detection of somatic point mutations in impure and heterogeneous cancer samples. Nat Biotechnol.

[53] McLaren W, Pritchard B, Rios D, Chen Y, Flicek P, Cunningham F (2010). Deriving the consequences of genomic variants with the Ensembl API and SNP Effect Predictor. Bioinformatics.

[54] http://www.broadinstitute.org/cancer/cga/indelocator.

[55] DePristo MA, Banks E, Poplin R, Garimella KV, Maguire JR, Hartl C, Philippakis AA, del Angel G, Rivas MA, Hanna M, McKenna A, Fennell TJ, Kernytsky AM (2011). A framework for variation discovery and genotyping using next-generation DNA sequencing data. Nat Genet.

[56] Lek M, Karczewski KJ, Minikel EV, Samocha KE, Banks E, Fennell T, O'Donnell-Luria AH, Ware JS, Hill AJ, Cummings BB, Tukiainen T, Birnbaum DP, Kosmicki JA (2016). Analysis of protein-coding genetic variation in 60,706 humans. Nature.

[57] Cerami E, Gao J, Dogrusoz U, Gross BE, Sumer SO, Aksoy BA, Jacobsen A, Byrne CJ, Heuer ML, Larsson E, Antipin Y, Reva B, Goldberg AP (2012). The cBio cancer genomics portal: an open platform for exploring multidimensional cancer genomics data. Cancer Discov.

[58] Choi Y, Sims GE, Murphy S, Miller JR, Chan AP (2012). Predicting the functional effect of amino acid substitutions and indels. PLoS One.

[59] Gao J, Aksoy BA, Dogrusoz U, Dresdner G, Gross B, Sumer SO, Sun Y, Jacobsen A, Sinha R, Larsson E, Cerami E, Sander C, Schultz N (2013). Integrative analysis of complex cancer genomics and clinical profiles using the cBioPortal. Sci Signal.

[60] Ng PC, Henikoff S (2001). Predicting deleterious amino acid substitutions. Genome Res.

[61] Reva B, Antipin Y, Sander C (2011). Predicting the functional impact of protein mutations: application to cancer genomics. Nucleic Acids Res.

